# Comparing the measurement properties of the EQ-5D-Y-3L, EQ-5D-Y-5L and CHU9D in children and adolescents: a measurement property study

**DOI:** 10.1007/s10198-025-01770-x

**Published:** 2025-04-12

**Authors:** Caique de Melo do Espirito Santo, Verônica Souza Santos, Yasmin Brasileiro de Souza, Aureliano Paolo Finch, Janine Verstraete, Gisela Cristiane Miyamoto, Tiê P. Yamato

**Affiliations:** 1 Master’s and Doctoral Program in Physical Therapy, Universidade Cidade de São Paulo, São Paulo, Brazil; 2 Physical Therapy Department, Universidade Cidade de São Paulo, São Paulo, Brazil; 3 EuroQol Research Foundation, Rotterdam, The Netherlands; 4 Department of Pediatrics and Child Health, Division of Pulmonology, University of Cape Town, Cape Town, South Africa; 5 Nepan Blue Mountains Local Health District, Penrith, NSW Australia; 6 School of Health Sciences, University of Sydney, Sydney, Australia

**Keywords:** Health-related quality of life, Musculoskeletal pain, Health status, Validation study, Psychometrics

## Abstract

**Background:**

The EQ-5D-Y-3L, EQ-5D-Y-5L and Child Health Utility 9-dimension (CHU9D) are instruments that measures health-related quality of life. These instruments are widely used in children and adolescents with health conditions, however the measurement properties of the three instruments have not been tested in Brazilian children.

**Objective:**

To compare and test the measurement properties of the EQ-5D-Y-3L, EQ-5D-Y-5L and CHU9D in Brazilian children and adolescents with and without any self-reported musculoskeletal pain.

**Methods:**

Children and adolescents aged 8–18 years were recruited from schools in Sao Paulo, Brazil and, self-completed the EQ-5D-Y-3L, EQ-5D-Y-5L and CHU9D at baseline and after 7 days. Reliability was determined by Kappa for the dimensions and intraclass correlation coefficient (ICC) for visual analogue scale (EQ VAS). Hypothesis were developed for construct validity and tested with Spearman and Pearson correlations (adequate if > 75% of the hypotheses confirmed). Children and adolescents with and without musculoskeletal pain were compared for known-group validity.

**Results:**

We included 356 children and adolescents, with 51% (*n* = 181) reporting musculoskeletal pain. Majority were male (53%) and mean age of 11.5 years (SD: 2.9). The EQ-5D-Y-3L, EQ-5D-Y-5L and CHU9D ranged from poor to moderate reliability. Reliability of the EQ VAS was substantial (ICC: 0.81, 95% confidence interval [CI]: 0.72 to 0.87) to moderate (ICC: 0.40, 95% CI: 0.24 to 0.53) for those with musculoskeletal pain and without pain, respectively. In those with musculoskeletal pain the association was weak to moderate, with > 75% of hypotheses confirmed, when comparing EQ-5D-Y-3L and EQ-D-Y-5L with the PedsQL™ and comparing EQ-5D-Y-5L with CHU9D. All instruments were able to discriminate those with and without musculoskeletal pain.

**Conclusion:**

All instruments had better measurement properties in children and adolescents with musculoskeletal pain, compared to those without for reliability and construct validity. These instruments could be used to assess health-related quality of life in Brazilian children and adolescents with musculoskeletal pain.

## Highlights

This study validated the EQ-5D-Y-3L, EQ-5D-Y-5L and CHU9D in Brazilian children and adolescents.

The measurement properties of the EQ-5D-Y-3L and EQ-5D-Y-5L had a better performance in children and adolescents with musculoskeletal pain compared those without musculoskeletal pain.

Clinicians and researchers can use the EQ-5D-Y-3L, EQ-5D-Y-5L, and CHU9D to measure health-related quality of life in children and adolescents.

## Introduction

Over the past two decades, there has been a growth in the development of health-related quality of life (HRQoL) instruments in children and adolescents [1]. Measuring HRQoL in children and adolescents is important to estimate the burden of different health conditions, for routine outcome measurement and to evaluate healthcare interventions [1, 2]. There are several generic HRQoL instruments for children and adolescents used as patient-reported outcome measures [1] including the Pediatric Quality of Life Inventory version 4.0 Generic Core Scales (PedsQL™) [3], KIDSCREEN [4], TNO-AZL Children’s Quality of Life (TACQOL) [5], Child Health Utility 9D (CHU9D) [6], EQ-5D-Y-3L and EQ-5D-Y-5L [7, 8]. The EQ-5D-Y-3L and CHU9D are the most commonly instruments used on measurement properties studies in children and adolescents [9, 10].

Musculoskeletal pain is a major cause of years lived with disability in children and adolescents aged 10–19 years [11]. The 1-month prevalence of musculoskeletal pain in children and adolescents in Brazil have been estimated as 27% and this condition can cause serious impact to general health and HRQoL [12, 13]. Furthermore, HRQoL is an important outcome of musculoskeletal pain in children and adolescents as it provides information on how the condition affects general health [1, 14]. To date, HRQoL in children with musculoskeletal pain has been investigated through instruments considering three main domains: physical, psychological and social [15, 16].

There are HRQoL instruments (known as generic preference-based measures) which can be used to estimate societal preference-weighted scores or utility [1, 9, 10]. Values range from 0 to 1, where 0 represents ‘dead’, and 1 represents ‘full health’ [17, 18]. Utility allows the calculation of the quality-adjusted life years (QALYs) [17, 19, 20], an outcome that combines the length of life and health related quality of life. The QALYs can be used for comparability between different interventions and health problems in economic evaluations [17, 19].

An important criterion for the selection of HRQoL measures, including generic preference-based measures, is whether the selected instruments show good measurement properties [21]. The EQ-5D-Y-3L, EQ-5D-Y-5L and CHU9D had their measurement properties tested previously in children and adolescents with different health conditions [22–28]. These instruments have never been tested in Brazilian children and adolescents. Therefore, the aim of this study was to compare and test the measurement properties of the EQ-5D-Y-3L, EQ-5D-Y-5L and CHU9D in Brazilian children and adolescents with and without any self-reported musculoskeletal pain.

## Methods

### Study design

This measurement study was conducted following the taxonomy and criteria of *COnsensus-based Standards for the selection of health Measurement INstruments* (COSMIN) [29, 30], and a checklist for judging preference-based measures of health that is used in economic evaluations (Supplementary information 1) [31]. This study was approved by the Human Ethics Committee of the *Universidade Cidade de São Paulo* (CAAE: 18752219.5.0000.0064). Inclusion of participants was dependent on signed informed consent from the parents or guardian and child assent.

### Instruments

#### Sociodemographic questionnaire

Children and adolescents self-completed the following sociodemographic information: age, sex, perception of the weight of their backpack and participation in sports and perception of relationship with their family. Their perceived relationship with their family included “how do you describe your relationship with your family?” with four response options (excellent, good, fair, and poor).

#### Presence and impact of pain in kids (PIP-Kids) questionnaire

The PIP-Kids questionnaire was originally developed for adolescents with low back pain [32]. The PIP-Kids has been shown to be valid and reliable in community context with Brazilian children and adolescents with musculoskeletal pain [33]. The questionnaire includes 10 questions that assess the presence and impact of pain in the last month. Questions 1 to 5 assess the presence of pain in the last month by body region, in relation to sports activities and, nature of pain (e.g., continuous and intermittent pain) over 3 months. Questions 6 to 10 assess the impact of pain with questions regarding health seeking behavior, medications usage, school absenteeism and interference with daily and physical activities [32, 33]. Responses are either “yes” or “no” with no summative score. The questionnaire was used to identify children and adolescents with musculoskeletal pain impacting theirs lives. Children and adolescents were considered as having musculoskeletal pain if they self-reported any pain in the back, neck, arms and/or legs in the last month, and that interfered with normal activities, recreational physical activities, or that cause school absenteeism in the last month [33]. Questions 1 and 8, 9 and/or 10 of the PIP-Kids questionnaire were used in this study to classify our sample with or without self-reported musculoskeletal pain. The English version of the PIP-Kids can be found in the Supplementary information 2.

#### Numerical rating scale pain (NRSP)

The NRSP ranging from 0 (no pain) to 10 (worst possible pain) was used for the participants to rate their perception on the pain intensity over the last month.

#### EQ-5D-Y instruments

The EQ-5D-Y-3L [7] and EQ-5D-Y-5L [8] are child-friendly versions of the EQ-5D developed by the EuroQol group for children and adolescents aged 8–15 years. Both instruments consist of two parts: the descriptive system and visual analogue scale (EQ VAS). The descriptive system has five dimensions of health: mobility (walking about), self-care (looking after myself), usual activities (doing usual activities), pain/discomfort (having pain or discomfort), and anxiety/depression (feeling worried, sad, or unhappy). Each EQ-5D-Y-3L dimension has three levels of severity: (1) no problems, (2) some problems, and (3) a lot of problems [7]. Similarly, the EQ-5D-Y-5L has five levels of severity: (1) no problems, (2) a little bit of problems, (3) some problems, (4) a lot of problems, and (5) cannot/extreme problems [8]. The participants further rate their perceived general health on the EQ VAS ranging from 0 to 100, where 0 represents the worst health imaginable and 100 represents the best health imaginable. The instruments have a recall period of “today”. Both instruments can generate a health state from the descriptive system (243 health states for the EQ-5D-Y-3L and 3,125 health states for the EQ-5D-Y-5L). The health state is coded by the combination of 5-digits representing the response level in each dimension. For example, the EQ-5D-Y-3L health states of 11,123 indicates no problems for “mobility”, “looking after myself” and “doing usual activities”, some problems for “having some pain or discomfort”, and a lot of problems for “feeling very worried, sad or unhappy”. Consequently, the EQ-5D instruments has three methods of interpretation: (1) the health state from the descriptive system; (2) the EQ VAS score representing self-rated health; and (3) the health state represented by the combination of 5-digits that can be converted into utility using the value set of a specific country [34]. The utilities represent the preferences for the health states of population ranging from 0 (dead) to 1 (full health) with negative values representing a specific health state named “worse than dead” [34]. The value set of the EQ-5D-Y-3L for Brazilian children and adolescents was recently published [35]. The official Brazilian-Portuguese EQ-5D-Y-3L and EQ-5D-Y-5L self-completed versions were used in this study.

#### Child health utility 9D (CHU9D)

The CHU9D is a generic HRQoL preference-based measures (self- and proxy-reported version) developed specifically for children and adolescents aged 7–17 years [6]. The instrument captures HRQoL for ‘today’ on nine dimensions: worry, sadness, pain, tiredness, annoyance, school, sleep, daily routine, and activities. Each dimension has five different levels representing increasing degrees of severity within each dimension (e.g., not worried, a little bit worried, a bit worried, quite worried, and very worried) [6]. The CHU9D can be interpreted by the descriptive system and the utility of each health state, which allows the calculation of QALYs for use in cost utility analysis. The utility of the CHU9D is available only for Australian adolescents [36], however, there is not utilities of this instrument available for any countries in America continent, including Brazil. The official Brazilian-Portuguese CHU9D self-completed version was used in this study.

#### Pediatric quality of life inventory version 4.0 generic core scales (PedsQL™)

The PedsQL™ generic self-report scales for children (8–12 years) and adolescents (13–18 years) were used to measure HRQoL [3, 15, 37]. The PedsQL™ includes 23 items across four domains: (1) physical functioning (eight items); (2) emotional functioning (five items); (3) social functioning (five items); and (4) school functioning (five items). Items are rated, with consideration of the past month, on a 5-point response scale: 0 = never a problem; 1 = almost never a problem; 2 = sometimes a problem; 3 = often a problem; 4 = almost always a problem. The item responses are reverse scored and summed with the final score ranging from 0 to 100, where higher scores indicate better HRQoL for total score, as well as the physical, emotional, social and school scores. Scores are not calculated if more than 50% of the items are missing. The Portuguese-Brazilian PedsQL™ version has been found to be valid and reliable in children and adolescents with rheumatic disease [15].

### Sample size calculation

The sample size calculation was based on the Chi-Square test for the known-group validity [38], as the sample size estimate was higher when compared to the reliability. For the known-group validity, we considered a power of 80%, an alpha of 0.05, and a dropout rate of 20%, in which a minimum sample of 350 children and adolescents was required. This further satisfies COSMIN recommendations for a sample of at least 100 participants for testing reliability, hypothesis testing for construct (convergent) validity and floor and ceiling effects [39].

### Participants and setting

We included children and adolescents aged 8 to 18 years old who could read and write Brazilian-Portuguese, from public and private schools in the urban area of Sao Paulo state. Children and adolescents with musculoskeletal pain was considered in those that self-reported any pain in the back, neck, arms and/or legs originating from the musculoskeletal system reported in the last month; and that interfered with normal activities, recreational physical activities, or that cause school absenteeism (also in the last month). All children and adolescents were screened on the PIP-Kids questionnaire [33]. Inclusion of the children and adolescents with musculoskeletal pain was considered those with a positive answer to the following questions: 1) “Did you feel some pain in back, neck, arms (included hands), or legs (included feet) in the last month?”; and at least one positive answer to: 8) “Did you miss school due to pain in your back, neck, arms, or legs in the last month?”, or 9) “Has your pain in your back, neck, arms, or legs interfered with your normal activities in the past month?”, and/or 10) “Does your pain in your back, neck, arms, or legs interfered your recreational activities (e.g., sport, walking, cycling, etc.) in the last month?” [33]. Pain due to traumas, sports injuries, surgery, or any specific condition (e.g., cancer, infection, fracture, inflammatory disease, or any diagnosis of the traumatic soft tissue injury - tendon and ligament) were excluded. Those children and adolescents without any musculoskeletal pain were considered those with a negative answer to the question 1 on PIP-Kids.

### Procedure

All children and adolescents received an explanation about the study and an envelope to take home. This envelope included a parental consent form and sociodemographic information. If the parents agreed for their children to take part in the study, the signed and completed forms (i.e., sociodemographic information) were returned to school within 7 days. For children with parental consent, assent was obtained from willing children and adolescents at school. At this point, children and adolescents were invited to answer the self-reported instruments (paper and pencil version), individually. Teachers and researcher members present in the classroom helped with interpretation if needed. The baseline assessment for children and adolescent self-completion included the following order: sociodemographic questionnaire, PIP-Kids [32], NRSP [40], EQ-5D-Y-5L [8], EQ-5D-Y-3L [41], CHU9D [6], and PedsQL™ [3, 15]. The order of administration of the EQ-5D-Y instruments were randomized to avoid ordering effects [42]. The same research packs, excluding the sociodemographic questionnaire and PedsQL™, were self-completed (in classroom) after 7-days, for the test-retest assessment of those children that were clinically stable. Change in health for test-retest reliability was classified according to the response on the PIP-Kids questionnaire between baseline and follow up.

### Data management and analysis

All the measurement properties (test-retest reliability, construct validity, known-group validity, feasibility) and ceiling and floor effects were tested and reported in two sets of children and adolescents: (1) with musculoskeletal pain, and (2) without any musculoskeletal pain. Descriptive analysis was performed for the participant’s sociodemographic characteristics. Mean and standard deviations or median and interquartile range were used for continuous variables with normal and non-normal distribution, respectively. Categorical variables were described with frequency and percentage. All data analyses were conducted using SPSS statistical package version 20 (IBM SPSS, Inc., Chicago, IL USA) and a significance was accepted at *p* ≤ 0.05.

#### Item response distribution

##### Feasibility

Feasibility of the EQ-5D-Y-3L, EQ-5D-Y-5L, EQ VAS, CHU9D, and PedsQL™ instruments was tested by calculating the percentage of missing data for each dimension of instruments. Responses that did not follow the instructions of the instrument were considered as missing data (e.g., two boxes selected for a single dimension or answers out the box). We calculated the missing data dividing the number of missing responses by the total number of participants for each dimension and then, multiplying by 100. The completion rate of the EQ-5D-Y-3L, EQ-5D-Y-5L, CHU9D, and PedsQL™ was tested. The completion rate of each instrument was calculated by subtracting the number of missing items of the instrument (from the total expected items) and then, dividing by the total expected items. The result was expressed as a percentage for each subsample of children and adolescents [31].

##### Ceiling and floor effects

Frequencies and proportion of reported problems in the descriptive system or, in the whole questionnaire were presented for the EQ-5D-Y-3L, EQ-5D-Y-5L, CHUD9, and PedsQL™. Ceiling and floor effects were calculated for each dimension of the descriptive system and by the health state of the descriptive system for the EQ-5D-Y-3L, EQ-5D-Y-5L (i.e., 11111), and CHU9D (i.e., 111111111). The reporting of the least and most problems was further analyzed by item on the EQ-5D-Y-3L, EQ-5D-Y-5L, CHU9D, and PedsQL™. For the health state of the EQ-5D youth instruments, the absolute and a percent reduction (ceiling_Y-3L_ – ceiling_Y-5L_ / ceiling_Y-3L_) was calculated for those with and without musculoskeletal [43, 44]. A ceiling or floor effect was considered in those reporting musculoskeletal pain if at least 15% of the participants reported the as lowest (e.g. 11111) or highest (e.g. 55555) score across all items respectively [39].

#### Measurement properties

##### Reliability (reliability and measurement error)

Repeat measures were administered after 7-days for test-retest reliability. Clinically stable was assumed if there was no change in the classification of pain according to the PIP-Kids between the baseline and retest. Reliability of the dimensions scores were assessed using the Kappa coefficient. Kappa coefficients were interpreted according to Landis and Koch guidelines as follows: <0.2 poor agreement, 0.21–0.40 fair agreement, 0,41 − 0.60 moderate agreement, 0.61–0.80 substantial agreement, and > 0.81 indicating almost perfect agreement [45]. Although previous studies presented different findings [23, 27, 41, 46–49], we expected a Kappa coefficient value < 0.70 for the dimensions of the descriptive system of the EQ-5D-Y-3L, EQ-5D-Y-5L, and CHU9D. The reliability of the EQ VAS was assessed using the Intraclass Correlation Coefficients – ICC (two-way mixed effects, absolute agreement, single rater/measurement) and 95% confidence intervals (95% CI). ICC was interpreted as: low reliability < 0.40, moderate reliability 0.40–0.75, substantial reliability 0.75–0.90, and excellent reliability > 0.90 [42]. Based on previous studies [41, 46, 50], we expected moderate to substantial reliability (ICC ≥ 0.40) for the EQ VAS of the EQ-5D-Y-3L and EQ-5D-Y-5L. Results were appraised according to COSMIN criteria in which the ICC ≥ 0.70 are considered adequate [39].

The measurement error was calculated by summing the number of agreements of the responses from descriptive systems between baseline and retest assessment. Then, we divided by the total of participants and multiplied by 100, for obtain the percentage of agreement for each dimension of the EQ-5D-Y-3L, EQ-5D-Y-5L, and CHU9D. Based on a previous study [41], we expected that the descriptive system of the EQ-5D-Y-3L, EQ-5D-Y-5L, and CHU9D instruments have an agreement > 70% in all dimensions. Measurement error was calculated by the Standard Error of Measurement (SEM) and the Smallest Detectable Change (SDC) for the EQ VAS of both instruments (EQ-5D-Y-3L and EQ-5D-Y-5L). For the SEM, we used the square root of error variance from ANOVA within-group analyses [39, 51]. The SDC was calculated using the formula “SDC = 1,96√2.SEM”. The ratio between the SEM and the instrument’s total score (EQ VAS) was used to indicate agreement as follow: very good agreement ≤ 5%, good agreement > 5% and ≤ 10%, doubtful agreement > 10% and ≤ 20%, and negative agreement > 20% [39, 42]. We expected that the EQ VAS for both instruments (EQ-5D-Y-3L and EQ-5D-Y-5L) have a very good to good agreement (SEM ≤ 5 to ≤ 10%). Although there are no specific previous studies with these instruments, our hypotheses are based on findings from previous studies on agreement specifically for the EQ-5D-3L instrument [52].

##### Hypothesis testing for construct validity

Hypotheses were developed a-priori to testing the construct validity regarding the strength of the correlations between the EQ-5D-Y-3L and EQ-5D-Y-5L with the PedsQL™ total scores [46] and CHU9D dimensions [53]. We also developed hypotheses of the correlation between the CHU9D and the PedsQL™ total scores [27, 28, 49]. All hypotheses were developed based on previous studies for the EQ-5D-Y-3L, EQ-5D-Y-5L and CHU9D considering the two subsamples of children and adolescents with and without any self-reported musculoskeletal pain [27, 28, 46, 49, 53]. The pre-specified hypotheses for the expected strength correlation between the instruments can be found in the Supplementary information 3 and 4. The correlations between scores were assessed with Spearman correlation coefficient (r) and the Pearson correlation coefficient for dimension comparison and comparison of continuous scores, respectively. The strength of the correlation was classified as follows: high (≥ 0.50), moderate (≥ 0.30 to < 0.50), and small (< 0.30) [54]. Results were appraised according to COSMIN criteria with construct validity considered as adequate if at least 75% of the results were in accordance with the pre-specified hypotheses [39].

##### Known-group validity

The known-group validity was performed to test the capacity of the EQ-5D-Y-3L, EQ-5D-Y-5L, and CHU9D discriminate between children and adolescents who reported musculoskeletal pain compared those without any musculoskeletal pain [55]. In those reporting musculoskeletal pain, we used the NRSP to assess the pain intensity and to classify the severity of the musculoskeletal pain: 0–3 (mild pain); 4–6 (moderate pain); 7–10 (severe pain) [40]. Thus, we also tested if the instruments were able to discriminate the severity of the musculoskeletal pain. Responses on the EQ-5D-Y-3L, EQ-5D-Y-5L, and CHU9D descriptive systems were compared by the Chi-Square test, and EQ VAS was compared by independent t-test or Mann-Whitney test [56].

We hypothesized a-priori that children and adolescents with self-reported musculoskeletal pain on the PIP-Kids, would report significantly more problems on the EQ-5D-Y-3L and EQ-5D-Y-5L dimensions of “having pain or discomfort”, “feeling worried, sad, or unhappy” as well as lower EQ VAS score compared to those without any musculoskeletal pain. We hypothesized that CHU9D items of “worry”, “sadness”, and “pain” would have a higher report of problems in children and adolescents with self-reported musculoskeletal pain compared those without any musculoskeletal pain. We hypothesized that children and adolescents with self-reported musculoskeletal pain with higher pain intensity (severe) on the NRSP, would report significantly more problems on the EQ-5D-Y-3L and EQ-5D-Y-5L dimensions of “having pain or discomfort”, “feeling worried, sad, or unhappy” and lower EQ VAS score. In addition, children and adolescents with higher pain intensity (severe) would report significantly more problems for “worry”, “sadness”, and “pain” in the CHU9D.

## Results

Of 1,760 envelopes that were sent out, 540 (31%) were returned with the signed consent to participate in the study. Of the responses returned, 356 (66%) children and adolescents met our inclusion criteria and were included for analysis. The sample included 181 (51%) children and adolescents who self-reported musculoskeletal pain in the last month. The mean age of the participants was 11.5 years old (standard deviation [SD]: 2.9). Of those with musculoskeletal pain, back pain (45.3%) and leg pain (35.9%) were the most common sites of pain, and the mean pain intensity was of 5.9 (SD: 3.0) points on the NRSP. Details of characteristics of the sample are in Table 1.

**Table 1:** Characteristics of the sample (*n* = 356)

	Total sample		
PIP-Kids questionnaire; n (%)^*a*^	*Q1 – Presence of pain*	181 (51.0)	
	*Q8 – Miss school*	83 (23.3)	
	*Q9 – Normal activities*	110 (30.9)	
	*Q10 – Recreational activities*	121 (24)	
Study sample	**MSK pain**	**Without pain**	**Total sample**
N (%)	181 (51)	175 (49)	356 (100)
Sex; n (%)			
*Male*	70 (38.7)	97 (55.4)	189 (53.1)
*Female*	111 (61.3)	78 (44.6)	167 (46.9)
Age; mean (SD)	11.6 (3.0)	11.5 (2.8)	11.5 (2.9)
Perception of the weight of backpack; n (%)^b, e^			
*Yes*	103 (57.9)	75 (43.6)	178 (51.0)
*No*	75 (42.1)	97 (56.4)	171 (49.0)
If yes, how heavy your backpack is?; n (%)^c, e^			
*Very heavy*	19 (18.5)	13 (17.3)	32 (18.0)
*Heavy*	52 (50.5)	32 (42.7)	84 (47.2)
*Not very heavy*	32 (31.0)	30 (40.0)	62 (34.8)
Relationship with your family; n (%)^d, e^			
*Excellent*	68 (38.2)	84 (48.6)	152 (43.3)
*Good*	82 (46.1)	84 (48.6)	166 (47.3)
*Fair*	28 (15.7)	5 (2.9)	33 (9.4)
*Poor*	0 (0)	0 (0)	0 (0)
Do you practice sports every week?; n (%)			
*Yes*	107 (59.1)	101 (58.0)	208 (58.6)
*No*	74 (40.9)	73 (41.7)	147 (41.3)
MSK pain?^¥^; n (%)			
*Back*	82 (45.3)	N/A	N/A
*Neck*	57 (31.5)	N/A	N/A
*Arms*	34 (18.8)	N/A	N/A
*Legs*	65 (35.9)	N/A	N/A
NRSP (0–10); mean (SD)	5.9 (3.0)	N/A	N/A

MSK: musculoskeletal pain; PIP-Kids: Presence and Impact of pain questionnaire; SD: standard deviation; NRSP: numerical rating scale pain [higher points indicate higher levels of pain intensity]; N/A: not applicable; n (%): absolute number and percentage

Q1: question 1 “Did you feel some pain in back, neck, arms [included hands] or legs [included feet] in the last month?”

Q8: question 8 “Did you miss school due to pain in your back, neck, arms or legs in the last month?”

Q9: question 9 “Has your pain in your back, neck, arms or legs interfered with your normal activities in the past month?

Q10: question 10 “Does your pain in your back, neck, arms or legs interfered your recreational activities [e.g., sport, walking, cycling, etc.] in the last month?”

^a^: Children and adolescents that answered ‘yes’ for the questions 1, 8, 9 and 10 in the PIP-Kids

^b^: Do you think your backpack is heavy?

^c^: If yes, how heavy do you think your backpack is?

^d^: How do you describe your relationship with your family?

^e^: Missing data ranged from 2 to 7 participants

^¥^: Children and adolescents could indicate pain in more than one site

### Item response distribution

#### Feasibility – missing responses

Considering the two subsamples of children and adolescents for each instrument, the percentage of missing data by dimension on the EQ-5D-Y-3L ranged from 0.6% (‘having pain or discomfort’) to 2.3% (‘looking after myself’). On the EQ-5D-Y-5L, the percentage of missing data by dimension ranged from 0% (‘mobility’, ‘looking after myself’ ‘and ‘feeling worried, sad, or unhappy’) to 12.7% (‘doing usual activities’). The percentage of missing data by dimension on the CHU9D varied from 1.7% (‘sleep’ and ‘daily routine’) to 5.7% (‘activities’), and on the PedsQL™ varied from 4.6% (‘hard to walk’) to 12.2% (‘sleep’). Furthermore, the completion rate was lowest for the PedsQL™ (91.1% for those with musculoskeletal pain and 92.1% for those without pain), followed by the CHU9D (96.6% for those without pain and 97.1% for those with musculoskeletal pain), the EQ-5D-Y-5L (96% for those with musculoskeletal pain and 98.5% for those without pain) and the EQ-5D-Y-3L (98.5% for those without pain and 98.7% for those with musculoskeletal pain). Details of the item response distribution for subsamples in the EQ-5D-Y-3L, EQ-5D-Y-5L, CHU9D and PedsQL™ are in the Table 2.

**Table 2:** Item response distribution in the EQ-5D-Y-3L, the EQ-5D-Y-5L, the CHU9D, and the pedsql™ from children and adolescents with (*n* = 181) and without (*n* = 175) musculoskeletal pain

	Level 1		Level 2		Level 3		Level 4		Level 5		Missing	
	MSK pain	Without pain	MSK pain	Without pain	MSK pain	Without pain	MSK pain	Without pain	MSK pain	Without pain	MSK pain	Without pain
	n (%)	n (%)	n (%)	n (%)	n (%)	n (%)	n (%)	n (%)	n (%)	n (%)	n (%)	n (%)
**EQ-5D-Y-3L**												
Mobility (walking about)	130 (71.8)	155 (88.6)	43 (23.8)	17 (9.7)	5 (2.8)	1 (0.6)	0 (0)	0 (0)	0 (0)	0 (0)	3 (1.7)	2 (1.1)
Looking after myself	158 (87.3)	166 (94.9)	15 (8.3)	3 (1.7)	6 (3.3)	2 (1.1)	0 (0)	0 (0)	0 (0)	0 (0)	2 (1.1)	4 (2.3)
Doing usual activities	117 (64.6)	147 (84.0)	51 (28.2)	24 (13.7)	11 (6.1)	1 (0.6)	0 (0)	0 (0)	0 (0)	0 (0)	2 (1.1)	3 (1.7)
Pain or discomfort	61 (33.7)	136 (77.7)	95 (52.5)	35 (20.0)	23 (12.7)	3 (1.7)	0 (0)	0 (0)	0 (0)	0 (0)	2 (1.1)	1 (0.6)
Worried, sad or unhappy	75 (41.4)	120 (68.6)	81 (44.8)	43 (24.6)	22 (12.2)	9 (5.1)	0 (0)	0 (0)	0 (0)	0 (0)	3 (1.7)	3 (1.7)
Health profile (‘11111’)	33 (18.2)	84 (48.0)										
Health profile (‘33333’)	2 (1.1)	1 (0.6)										
EQ VAS (SD)	76.1 (23.8)	89.8 (17.3)									8 (4.4)	8 (4.6)
**EQ-5D-Y-5L**												
Mobility (walking about)	121 (66.9)	155 (88.6)	43 (23.8)	14 (8.0)	6 (3.3)	3 (1.7)	5 (2.8)	1 (0.6)	3 (1.7)	2 (1.1)	3 (1.7)	0 (0)
Looking after myself	154 (85.1)	165 (94.3)	13 (7.2)	7 (4.0)	4 (2.2)	1 (0.6)	3 (1.7)	1 (0.6)	4 (2.2)	1 (0.6)	3 (1.7)	0 (0)
Doing usual activities	100 (55.2)	141 (80.6)	36 (19.9)	15 (8.6)	17 (9.4)	5 (2.9)	1 (0.6)	1 (0.6)	4 (2.2)	1 (0.6)	23 (12.7)	12 (6.9)
Pain or discomfort	65 (35.9)	126 (72)	61 (33.7)	36 (20.6)	27 (14.9)	6 (3.4)	17 (9.4)	3 (1.7)	8 (4.4)	3 (1.7)	3 (1.7)	1 (0.6)
Worried, sad or unhappy	68 (37.6)	120 (68.6)	59 (32.6)	35 (20.0)	23 (12.7)	14 (8.0)	8 (4.4)	4 (2.3)	19 (10.5)	2 (1.1)	4 (2.2)	0 (0)
Health profile (‘11111’)	29 (16.0)	82 (46.9)										
Health profile (‘55555’)	1 (0.6)	1 (0.6)										
EQ VAS (SD)	75.7 (25.3)	89.3 (17.4)									10 (5.5)	7 (4.0)
**CHU9D**												
Worried	84 (46.4)	121 (69.1)	59 (32.6)	32 (18.3)	13 (7.2)	8 (4.6)	13 (7.2)	3 (1.7)	7 (3.9)	2 (1.1)	5 (2.8)	9 (5.1)
Sad	86 (47.5)	123 (70.3)	51 (28.2)	29 (16.6)	18 (9.9)	5 (2.9)	7 (3.9)	6 (3.4)	13 (7.2)	3 (1.7)	6 (3.3)	9 (5.1)
Pain	59 (32.6)	128 (73.1)	77 (42.5)	30 (17.1)	20 (11.0)	5 (2.9)	16 (8.8)	4 (2.3)	0 (0)	0 (0)	9 (5.0)	8 (4.6)
Tired	47 (26.0)	84 (48.0)	74 (40.9)	63 (36.0)	15 (8.3)	11 (6.3)	18 (9.9)	7 (4.0)	23 (12.7)	5 (2.9)	4 (2.2)	5 (2.9)
Annoyed	107 (59.1)	135 (77.1)	32 (17.7)	24 (13.7)	16 (8.8)	7 (4.0)	5 (2.8)	3 (1.7)	16 (8.8)	3 (1.7)	5 (2.8)	3 (1.7)
Schoolwork/homework	102 (56.4)	138 (78.9)	47 (26.0)	25 (14.3)	12 (6.6)	7 (4.0)	9 (5.0)	1 (0.6)	5 (2.8)	0 (0)	6 (3.3)	4 (2.3)
Sleep	79 (43.6)	130 (74.3)	53 (29.3)	27 (15.4)	17 (9.4)	6 (3.4)	12 (6.6)	7 (4.0)	17 (9.4)	2 (1.1)	3 (1.7)	3 (1.7)
Daily routine	126 (69.6)	147 (84.0)	27 (14.9)	17 (9.7)	14 (7.7)	5 (2.9)	4 (2.2)	0 (0)	6 (3.3)	3 (1.7)	4 (2.2)	3 (1.7)
Activities	105 (58.0)	132 (75.4)	47 (26.0)	23 (13.1)	12 (6.6)	7 (4.0)	5 (2.8)	2 (1.1)	6 (3.3)	1 (0.6)	6 (3.3)	10 (5.7)
Health profile (‘111111111’)	10 (5.5)	34 (19.4)										
Health profile (‘555555555’)	0 (0)	0 (0)										
**PedsQL™**												
Hard to walk	109 (60.2)	135 (77.1)	28 (15.5)	18 (10.3)	18 (9.9)	9 (5.1)	8 (4.4)	3 (1.7)	8 (4.4)	2 (1.1)	10 (5.5)	8 (4.6)
Hard to run	72 (39.8)	126 (72.0)	37 (20.4)	15 (8.6)	31 (17.1)	15 (8.6)	15 (8.3)	6 (3.4)	14 (7.7)	2 (1.1)	12 (6.6)	11 (6.3)
Play sports/do activity physical	86 (47.5)	125 (71.4)	36 (19.9)	21 (12.0)	26 (14.4)	10 (5.7)	10 (5.5)	1 (0.6)	12 (6.6)	3 (1.7)	11 (6.1)	15 (8.6)
Lift something heavy	56 (30.9)	83 (47.4)	30 (16.6)	30 (17.1)	48 (26.5)	33 (18.9)	17 (9.4)	6 (3.4)	18 (9.9)	11 (6.3)	12 (6.6)	12 (6.9)
Take a bath/shower myself	152 (84.0)	153 (87.4)	6 (3.3)	5 (2.9)	4 (2.2)	1 (0.6)	6 (3.3)	1 (0.6)	3 (1.7)	3 (1.7)	10 (5.5)	12 (6.9)
To do household chores	90 (49.7)	129 (73.7)	37 (20.4)	14 (8.0)	20 (11.0)	9 (5.1)	8 (4.4)	5 (2.9)	9 (5.0)	5 (2.9)	17 (9.4)	13 (7.4)
Pain/aches	32 (17.7)	83 (47.4)	19 (10.5)	35 (20.0)	51 (28.2)	35 (20.0)	33 (18.2)	3 (1.7)	25 (13.8)	3 (1.7)	21 (11.6)	16 (9.1)
Low energy levels	62 (34.3)	104 (59.4)	29 (16.0)	29 (16.6)	37 (20.4)	20 (11.4)	16 (8.8)	5 (2.9)	20 (11.0)	4 (2.3)	17 (9.4)	13 (7.4)
Afraid or scared	51 (28.2)	81 (46.3)	33 (18.2)	33 (18.9)	38 (21.0)	29 (16.6)	17 (9.4)	7 (4.0)	22 (12.2)	11 (6.3)	20 (11.0)	14 (8.0)
Sad	43 (23.8)	82 (46.9)	31 (17.1)	37 (21.1)	39 (21.5)	26 (14.9)	23 (12.7)	4 (2.3)	26 (14.4)	10 (5.7)	19 (10.5)	16 (9.1)
Angry	25 (13.8)	60 (34.3)	22 (12.2)	30 (17.1)	40 (22.1)	39 (22.3)	38 (21.0)	15 (8.6)	38 (21.0)	16 (9.1)	18 (9.9)	15 (8.6)
Sleep	50 (27.6)	91 (52.0)	26 (14.4)	30 (17.1)	38 (21.0)	26 (14.9)	21 (11.6)	8 (4.6)	24 (13.3)	6 (3.4)	22 (12.2)	14 (8.0)
Worry about what will happen to me	34 (18.8)	54 (30.9)	21 (11.6)	31 (17.7)	34 (18.8)	35 (20.0)	28 (15.5)	21 (12.0)	45 (24.9)	18 (10.3)	19 (10.5)	16 (9.1)
Getting along with others	75 (41.4)	105 (60.0)	30 (16.6)	26 (14.9)	29 (16.0)	22 (12.6)	19 (10.5)	6 (3.4)	13 (7.2)	5 (2.9)	15 (8.3)	11 (6.3)
Others don’t what to be my friend	71 (39.2)	106 (60.6)	44 (24.3)	36 (20.6)	27 (14.9)	13 (7.4)	11 (6.1)	2 (1.1)	13 (7.2)	1 (0.6)	15 (8.3)	17 (9.7)
Others tease me	74 (40.9)	117 (66.9)	41 (22.7)	22 (12.6)	24 (13.3)	13 (7.4)	9 (5.0)	5 (2.9)	19 (10.5)	4 (2.3)	14 (7.7)	14 (8.0)
Cannot do thing others my age can	71 (39.2)	107 (61.1)	43 (23.8)	28 (16.0)	27 (14.9)	17 (9.7)	13 (7.2)	7 (4.0)	12 (6.6)	1 (0.6)	15 (8.3)	15 (8.6)
Hard to keep up with other	83 (45.9)	105 (60.0)	33 (18.2)	30 (17.1)	27 (14.9)	18 (10.3)	8 (4.4)	3 (1.7)	15 (8.3)	4 (2.3)	15 (8.3)	15 (8.6)
Hard to pay attention in class	56 (30.9)	94 (53.7)	30 (16.6)	26 (14.9)	41 (22.7)	30 (17.1)	18 (9.9)	6 (3.4)	18 (9.9)	6 (3.4)	18 (9.9)	13 (7.4)
Forget things	21 (11.6)	46 (26.3)	27 (14.9)	37 (21.1)	46 (25.4)	36 (20.6)	36 (19.9)	23 (13.1)	33 (18.2)	17 (9.7)	18 (9.9)	16 (9.1)
Trouble keeping up with schoolwork	63 (34.8)	102 (58.3)	29 (16.0)	27 (15.4)	36 (19.9)	18 (10.3)	17 (9.4)	5 (2.9)	18 (9.9)	8 (4.6)	18 (9.9)	15 (8.6)
Missing class (not feeling well)	54 (29.8)	78 (44.6)	43 (23.8)	42 (24.0)	38 (21.0)	28 (16.0)	12 (6.6)	6 (3.4)	17 (9.4)	5 (2.9)	17 (9.4)	16 (9.1)
Missing class (doctor/hospital)	61 (33.7)	80 (45.7)	50 (27.6)	37 (21.1)	32 (17.7)	34 (19.4)	7 (3.9)	4 (2.3)	15 (8.3)	7 (4.0)	16 (8.8)	13 (7.4)
PedsQL™ total score (SD)	65.9 (19.6)	81.4 (13.0)									15 (8.3)	12 (6.9)

MSK: musculoskeletal pain; EQ VAS: visual analogue scale; SD: standard deviation; CHU9D: Child Health Utility 9D; PedsQL™: The Pediatric Quality of Life Inventory™ Version 4.0; n (%): Absolute number and percentage

*EQ VAS [0-100]: higher points indicate better health-related quality of life*

*PedsQL™ total score: [0-100]: higher points indicate better health-related quality of life*

*EQ-5D-Y-5L: levels 1*,* 2*,* 3*,* 4*,* and 5 represent ‘no problems’*,* ‘a little bit of a problem’*,* ‘some problems’*,* ‘a lot of problems’ and*,* ‘extreme problems’ for responses for the descriptive system*

*EQ-5D-Y-3L: levels 1*,* 2*,* and 3 represent ‘no problems’*,* ‘some problems’*,* and ‘a lot of problems’*

*CHU9D: levels 1*,* 2*,* 3*,* 4*,* and 5 represent (i.e.*,* worried) ‘not worried’*,* ‘a little bit worried’*,* ‘a bit worried’*,* ‘quite worried’*,* and ‘very worried’*

*PedsQL™: levels 1*,* 2*,* 3*,* 4*,* and 5 represent ‘never’*,* ‘almost never’*,* ‘sometimes’*,* ‘often’*,* ‘almost always’*

#### Ceiling and floor effects

Table 2 shows the distribution responses to all instruments for children and adolescents. When considering those with musculoskeletal pain, the ceiling effect (11111) reduced on the EQ-5D-Y-5L (16.0%) when compared to the EQ-5D-Y-3L (18.2%). The CHU9D had a low reporting of no problems across all dimensions (111111111) in those with musculoskeletal pain (5.5%). The item response distributions of all instruments for total sample can be found in the Supplementary material 5. The proportions of “no problems” responses for the EQ-5D-Y-3L and EQ-5D-Y-5L, and ceiling effect reduction for both subsamples and total sample, can be found in the Supplementary material 6.

### Measurement properties

#### Test-retest reliability

Two hundred and thirty-one children were classified as clinically stable based on their report on the PIP-Kids (*n* = 96 for those with musculoskeletal pain and *n* = 135 for those without musculoskeletal pain) between baseline and 7-days retest after baseline (Table 3). Considering both subsamples, Kappa coefficients ranged from fair to moderate reliability for the descriptive system for EQ-5D-Y-3L (0.25 to 0.48) and EQ-5D-Y-5L (0.20 to 0.49) whereas CHU9D ranged from poor to moderate reliability for the descriptive system (0.11 to 0.46). Considering both subsamples, the agreement of the descriptive system ranged from 58.3 to 93.4% for EQ-5D-Y-3L; from 41.7 to 94.9% for EQ-5D-Y-5L; and from 41.7 to 83.2% for CHU9D. For children and adolescents with musculoskeletal pain, the ICC of the EQ VAS for EQ-5D-Y-3L and EQ-5D-Y-5L was of 0.81 (95% CI: 0.72 to 0.87) and 0.80 (95% CI: 0.71 to 0.86), respectively, indicating substantial reliability. For those without musculoskeletal pain, the ICC of the EQ VAS for the EQ-5D-Y-3L and EQ-5D-Y-5L was of 0.40 (95% CI: 0.24 to 0.53), and 0.58 (95% CI: 0.45 to 0.68), respectively, indicating moderate reliability (Table 3). Details of reliability and measurement error for both subsamples are described in Table 3 and, for the total sample, data can be found in Supplementary material 7.

**Table 3:** Test-retest reliability and measurement error of the EQ-5D-Y-5L, the EQ-5D-Y-3L and the CHU9D in a clinically stable sample of children and adolescents with and without musculoskeletal pain

	EQ-5D-Y-5L									EQ-5D-Y-3L			
**Dimensions**	Musculoskeletal pain (*n* = 96)				Without musculoskeletal pain (*n* = 135)					Musculoskeletal pain (*n* = 96)		Without musculoskeletal pain (*n* = 135)	
	Kappa	Agreement (%)			Kappa			Agreement (%)		Kappa	Agreement (%)	Kappa	Agreement (%)
Mobility (walking about)	0.30	61.5			0.27			87.6		0.39	71.9	0.25	86.1
Looking after myself	0.49	82.3			0.36			94.9		0.47	84.4	0.32	93.4
Doing usual activities	0.28	49.0			0.26			75.2		0.34	64.6	0.27	83.9
Having pain or discomfort	0.20	41.7			0.33			75.2		0.32	58.3	0.32	78.1
Feeling worried, sad or unhappy	0.35	51.0			0.37			74.5		0.48	63.5	0.47	77.4
ICC of the EQ VAS (95% CI)	0.80 (0.71 to 0.86)				0.58 (0.45 to 0.68)					0.81 (0.72 to 0.87)		0.40 (0.24 to 0.53)	
SEM (%) of the EQ VAS	14.7				9.7					14.9		10.4	
SDC of the EQ VAS	10.6				8.6					10.7		8.9	
**Dimensions**	**CHU9D**												
	Musculoskeletal pain (*n* = 96)					Without musculoskeletal pain (*n* = 135)							
	Kappa			Agreement (%)		Kappa			Agreement (%)				
Worried	0.39		57.3		0.20		66.4						
Sad	0.27		47.9		0.39		74.5						
Pain	0.22		41.7		0.11		67.9						
Tired	0.35		52.1		0.34		62.0						
Annoyed	0.28		51.0		0.29		77.4						
Schoolwork	0.30		56.3		0.41		82.5						
Sleep	0.30		49.0		0.22		69.3						
Daily routine	0.46		69.8		0.12		83.2						
Activities	0.40		59.4		0.30		75.9						

ICC: intraclass correlation coefficient [type two-way mixed effects]; EQ VAS: EQ visual analogue scale; SEM: standard error of measurement; SDC: smallest detectable change; CHU9D: Child Health Utility 9 Dimension; Kappa: kappa coefficient; 95% CI: confidence interval of 95%; n: number of children and adolescents

#### Hypothesis testing for construct validity

For those with musculoskeletal pain, the hypotheses on the EQ-5D-Y-3L and EQ-5D-Y-5L compared to the PedsQL™ showed small to moderate correlations. This satisfied 89% and 100% of our pre-specified hypotheses for the EQ-5D-Y-3L and EQ-5D-Y-5L, respectively, indicating adequate construct validity (Table 4). For those without musculoskeletal pain, the hypotheses on the EQ-5D-Y-3L and EQ-5D-Y-5L compared to the PedsQL™ were small to moderate. However, only accounted for 33% of our pre-specified hypotheses for both instruments, indicating inadequate validity (Table 4). The EQ-5D-Y-3L showed small to high correlations compared to the CHU9D for both subsamples. However, only 44% and 69% of the pre-specified hypotheses were in accordance with children and adolescents with and without musculoskeletal pain, respectively, indicating inadequate validity (Table 4). On the other hand, the EQ-5D-Y-5L demonstrated small to high correlations compared to the CHU9D for both subsamples. This reached 81% and 75% of our pre-specified hypotheses for children and adolescents with and without musculoskeletal pain, respectively, indicating adequate construct validity between the EQ-5D-Y-5L compared to the CHU9D (Table 4). The strength of correlations between of the EQ-5D-Y-3L and EQ-5D-Y-5L compared to the CHU9D and the PedsQL™ is available in supplementary material 8.

**Table 4:**
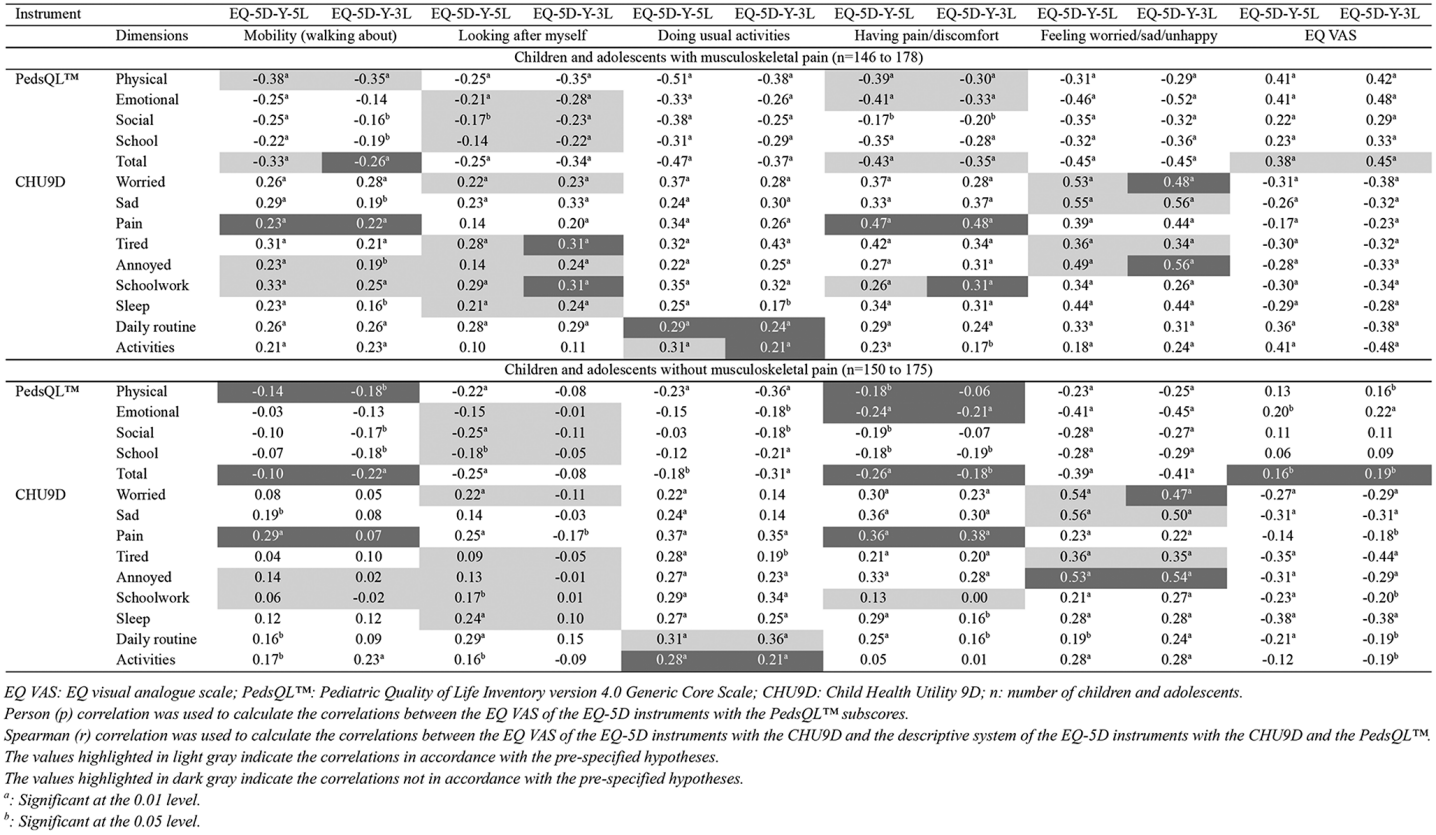
Strength of correlations between the EQ-5D-Y-3L and the EQ-5D-Y-5L compared to the CHU9D and the pedsql™ subscores of the two subsamples

The CHU9D showed small to high correlations compared to the PedsQL™ for both subsamples. However, only 49% and 51% of the pre-specified hypotheses were in accordance with children and adolescents with and without musculoskeletal pain, respectively, indicating inadequate validity (Table 5). The strength of correlations between of the CHU9D compared to the PedsQL™ for total sample is available in supplementary material 9.

**Table 5:**
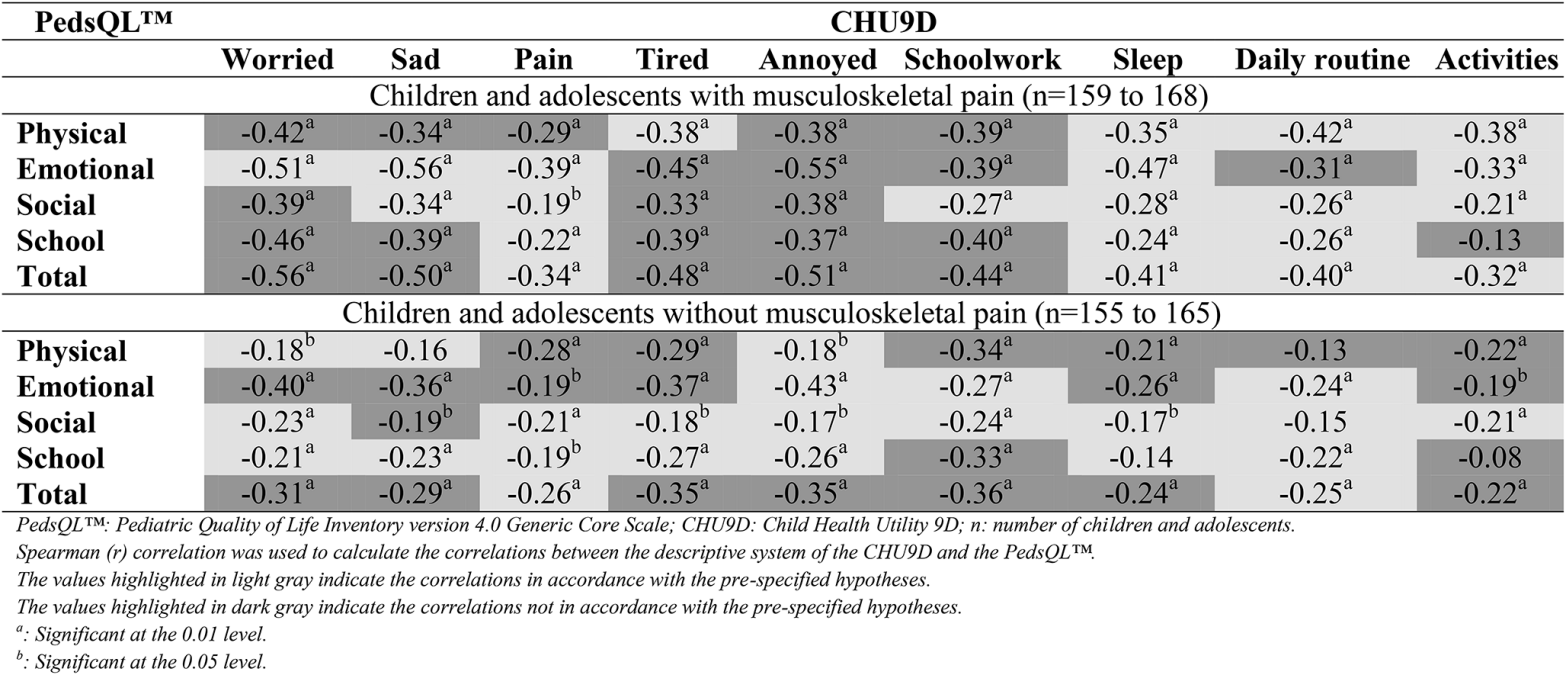
Strength of correlations between the CHU9D and the pedsql™ subscores of the two subsamples

#### Known-group validity

Children and adolescents with musculoskeletal pain reported significantly (*p* < 0.05) more problems compared to those without musculoskeletal pain, for all dimensions of the descriptive system of the EQ-5D-Y-3L, EQ-5D-Y-5L (except for the ‘looking after myself’ dimension), EQ VAS and CHU9D (Table 6). Children and adolescents with higher pain intensity reported significantly (*p* < 0.05) more problems on the descriptive system for the EQ-5D-Y-3L and EQ-5D-Y-5L (except for the ‘looking after myself’ and ‘doing usual activities’ dimensions). Furthermore, the EQ VAS mean was significantly lower than those children and adolescents with higher pain intensity, as measured on the NSRP for the EQ-5D-Y-3L and EQ-5D-Y-5L. Children and adolescents with higher pain intensity reported significantly more problems only for “worried”, “pain” and “tired” dimensions in the CHU9D. Comparison of the known-group validity for the children and adolescents with musculoskeletal pain according to the severity of pain on the EQ-5D-Y-3L, EQ-5D-Y-5L, and CHU9D are shown in Table 7.

**Table 6:** Comparison of the known-group validity for both samples of children and adolescents with (*n* = 181) and without (*n* = 175) musculoskeletal pain for the CHU9D, the EQ-5D-Y-5L, and the EQ-5D-Y-3L

	MSK pain	Without MSK pain		MSK pain	Without MSK pain		MSK pain	Without MSK pain
	n (%)	n (%)		n (%)	n (%)		n (%)	n (%)
**CHU9D**			**EQ-5D-Y-5L**			**EQ-5D-Y-3L**		
Worried*	*n* = 176	*n* = 166	Mobility (walking about)*	*n* = 178	*n* = 175	Mobility (walking about)*	*n* = 178	*n* = 173
No worried	84 (47.7)	121 (72.9)	No problems	121 (68.0)	155 (88.6)	No problems	130 (73)	155 (89.6)
A little bit worried	59 (33.5)	32 (19.3)	A little bit of problems	43 (24.2)	14 (8.0)	Some problems	43 (24.2)	17 (9.8)
A bit worried	13 (7.4)	8 (4.8)	Some problems	6 (3.4)	3 (1.7)	A lot of problems	5 (2.8)	1 (0.6)
Quite worried	13 (7.4)	3 (1.8)	A lot of problems	5 (2.8)	1 (0.6)	Looking after myself*	*n* = 179	*n* = 171
Very worried	7 (4.0)	2 (1.2)	Extreme problems	3 (1.7)	2 (1.1)	No problems	158 (88.3)	166 (97.1)
Sad*	*n* = 175	*n* = 166	Looking after myself	*n* = 178	*n* = 175	Some problems	15 (8.4)	3 (1.8)
No sad	86 (49.1)	123 (74.1)	No problems	154 (86.5)	165 (94.3)	A lot of problems	6 (3.4)	2 (1.2)
A little bit sad	51 (29.1)	29 (17.5)	A little bit of problems	13 (7.3)	7 (4.0)	Doing usual activities*	*n* = 179	*n* = 172
A bit sad	18 (10.3)	5 (3.0)	Some problems	4 (2.2)	1 (0.6)	No problems	117 (65.4)	147 (85.5)
Quite sad	7 (4.0)	6 (3.6)	A lot of problems	3 (1.7)	1 (0.6)	Some problems	51 (28.5)	24 (14.0)
Very sad	13 (7.4)	3 (1.8)	Extreme problems	4 (2.2)	1 (0.6)	A lot of problems	11 (6.1)	1 (0.6)
Pain*	*n* = 172	*n* = 167	Doing usual activities*	*n* = 158	*n* = 163	Having pain or discomfort*	*n* = 179	*n* = 174
No pain	59 (34.3)	128 (76.6)	No problems	100 (63.3)	141 (86.5)	No problems	61 (34.1)	136 (78.2)
A little bit pain	77 (44.8)	30 (18.0)	A little bit of problems	36 (22.8)	15 (9.2)	Some problems	95 (53.1)	35 (20.1)
A bit pain	20 (11.6)	5 (3.0)	Some problems	17 (10.8)	5 (3.1)	A lot of problems	23 (12.8)	3 (1.7)
Quite pain	16 (9.3)	4 (2.4)	A lot of problems	1 (0.6)	1 (0.6)	Feeling worried, sad or unhappy*	*n* = 178	*n* = 172
Very pain	0 (0)	0 (0)	Extreme problems	4 (2.5)	1 (0.6)	No problems	75 (42.1)	120 (69.8)
Tired*	*n* = 177	*n* = 170	Having pain or discomfort*	*n* = 178	*n* = 174	Some problems	81 (45.5)	43 (25)
No tired	47 (26.5)	84 (49.4)	No problems	65 (36.5)	126 (72.4)	A lot of problems	22 (12.4)	9 (5.2)
A little bit tired	74 (41.8)	63 (37.0)	A little bit of problems	61 (34.3)	36 (20.7)	EQ VAS, median (IQR)*	85 (40)	100 (12)
A bit tired	15 (8.5)	11 (6.5)	Some problems	27 (15.2)	6 (3.4)			
Quite tired	18 (10.2)	7 (4.1)	A lot of problems	17 (9.6)	3 (1.7)			
Very tired	23 (13.0)	5 (2.9)	Extreme problems	8 (4.5)	3 (1.7)			
Annoyed*	*n* = 176	*n* = 172	Feeling worried, sad or unhappy*	*n* = 177	*n* = 175			
No annoyed	107 (60.8)	135 (78.5)	No problems	68 (38.4)	120 (68.6)			
A little bit annoyed	32 (18.2)	24 (13.9)	A little bit of problems	59 (33.3)	35 (20.0)			
A bit annoyed	16 (9.1)	7 (4.1)	Some problems	23 (13.0)	14 (8.0)			
Quite annoyed	5 (2.8)	3 (1.7)	A lot of problems	8 (4.5)	4 (2.3)			
Very annoyed	16 (9.1)	3 (1.7)	Extreme problems	19 (10.7)	2 (1.1)			
Schoolwork/homework*	*n* = 175	*n* = 171	EQ VAS, median (IQR)*	85 (45)	97 (14)			
No problems	102 (58.3)	138 (80.7)						
A little bit problems	47 (26.8)	25 (14.6)						
A bit problems	12 (6.9)	7 (4.1)						
Quite problems	9 (5.1)	1 (0.6)						
Very problems	5 (2.9)	0 (0)						
Sleep*	*n* = 178	*n* = 172						
No problems	79 (44.4)	130 (75.5)						
A little bit problems	53 (29.8)	27 (15.7)						
A bit problems	17 (9.5)	6 (3.5)						
Quite problems	12 (6.7)	7 (4.1)						
Very problems	17 (9.5)	2 (1.2)						
Daily routine*	*n* = 177	*n* = 172						
No problems	126 (71.2)	147 (85.5)						
A little bit problems	27 (15.2)	17 (9.9)						
A bit problems	14 (7.9)	5 (2.9)						
Quite problems	4 (2.3)	0 (0)						
Very problems	6 (3.4)	3 (1.7)						
Able activities*	*n* = 175	*n* = 165						
No problems	105 (60.0)	132 (80.0)						
A little bit problems	47 (26.9)	23 (13.9)						
A bit problems	12 (6.9)	7 (4.2)						
Quite problems	5 (2.8)	2 (1.2)						
Very problems	6 (3.4)	1 (0.6)						

MSK: musculoskeletal; EQ VAS: EQ visual analogue scale; CHU9D: Child Health Utility 9-Dimension; IQR: interquartile range; n: number of children and adolescents

n (%): absolute number and percentage

^*^ p-value < 0.05 (Chi-square for the descriptive system and Mann-Whitney for the EQ visual analogue scale)

**Table 7:** Comparison of the known-group validity for children and adolescents with musculoskeletal pain according to the severity pain on the CHU9D, the EQ-5D-Y-5L, and the EQ-5D-Y-3L

	MSK pain				MSK pain				MSK pain		
	Mild pain	Moderate pain	Severe pain		Mild pain	Moderate pain	Severe pain		Mild pain	Moderate pain	Severe pain
	n (%)	n (%)	n (%)		n (%)	n (%)	n (%)		n (%)	n (%)	n (%)
**CHU9D**				**EQ-5D-Y-5L**				**EQ-5D-Y-3L**			
Worried*	*n* = 36	*n* = 61	*n* = 74	Mobility*	*n* = 37	*n* = 60	*n* = 75	Mobility*	*n* = 37	*n* = 61	*n* = 74
No worried	23 (28.4)	24 (29.6)	34 (42)	No problems	35 (94.6)	42 (70)	39 (52)	No problems	33 (89.2)	45 (73.8)	47 (63.5)
A little bit worried	8 (14)	24 (42.1)	25 (43.9)	A little bit of problems	2 (5.4)	15 (25)	25 (33.3)	Some problems	4 (10.8)	16 (26.2)	23 (31.3)
A bit worried	4 (30.8)	5 (38.5)	4 (30.8)	Some problems	0 (0)	3 (5)	3 (4)	A lot of problems	0 (0)	0 (0)	4 (5.4)
Quite worried	0 (0)	8 (61.5)	5 (38.5)	A lot of problems	0 (0)	0 (0)	5 (6.7)	Looking after myself	*n* = 37	*n* = 61	*n* = 75
Very worried	1 (14.3)	0 (0)	6 (85.7)	Extreme problems	0 (0)	0 (0)	3 (4)	No problems	37 (100)	55 (90.2)	61 (81.3)
Sad	*n* = 36	*n* = 61	*n* = 73	Looking after myself	*n* = 37	*n* = 60	*n* = 75	Some problems	0 (0)	4 (6.6)	10 (13.3)
No sad	25 (30.5)	28 (34.1)	29 (35.4)	No problems	34 (91.9)	53 (88.3)	62 (82.7)	A lot of problems	0 (0)	2 (3.3)	4 (5.3)
A little bit sad	10 (20)	17 (34)	23 (46)	A little bit of problems	1 (2.7)	4 (6.7)	12 (7)	Doing usual activities	*n* = 37	*n* = 61	*n* = 75
A bit sad	1 (5.6)	8 (44.4)	9 (50)	Some problems	1 (2.7)	1 (1.7)	4 (2.3)	No problems	29 (78.4)	41 (67.2)	42 (56)
Quite sad	0 (0)	4 (57.1)	3 (42.9)	A lot of problems	0 (0)	0 (0)	3 (1.7)	Some problems	6 (16.2)	18 (29.5)	27 (36)
Very sad	0 (0)	4 (30.8)	9 (69.2)	Extreme problems	1 (2.7)	2 (3.3)	4 (2.3)	A lot of problems	2 (5.4)	2 (3.3)	6 (8)
Pain*	*n* = 36	*n* = 60	*n* = 71	Doing usual activities	*n* = 32	*n* = 54	*n* = 68	Having pain or discomfort*	*n* = 37	*n* = 61	*n* = 75
No pain	20 (37)	13 (24.1)	21 (38.9)	No problems	27 (84.4)	35 (64.8)	35 (51.5)	No problems	27 (73.0)	14 (23)	16 (21.3)
A little bit pain	16 (20.8)	36 (46.8)	25 (32.5)	A little bit of problems	4 (12.5)	13 (24.1)	19 (27.9)	Some problems	10 (27)	41 (67.2)	42 (56)
A bit pain	0 (0)	7 (35)	13 (65)	Some problems	1 (3.1)	5 (9.3)	10 (14.7)	A lot of problems	0 (0)	6 (9.8)	17 (22.7)
Quite pain	0 (0)	4 (25)	12 (75)	A lot of problems	0 (0)	0 (0)	1 (1.5)	Feeling worried, sad or unhappy*	*n* = 37	*n* = 61	*n* = 74
Very pain	0 (0)	0 (0)	0 (0)	Extreme problems	0 (0)	1 (1.9)	3 (4.4)	No problems	26 (70.3)	25 (41)	20 (27)
Tired*	*n* = 37	*n* = 61	*n* = 73	Having pain or discomfort*	*n* = 37	*n* = 60	*n* = 75	Some problems	11 (29.7)	30 (49.2)	39 (52.7)
No tired	18 (42.9)	12 (28.6)	12 (28.6)	No problems	27 (73)	15 (25)	18 (24)	A lot of problems	0 (0)	6 (9.8)	15 (20.3)
A little bit tired	15 (20.3)	28 (37.8)	31 (41.9)	A little bit of problems	8 (21.6)	25 (41.7)	27 (36)	EQ VAS*	*n* = 36	*n* = 59	*n* = 76
A bit tired	2 (14.3)	8 (57.1)	4 (28.6)	Some problems	2 (5.4)	12 (20)	13 (17.3)	Mean (SD)	87.3 (13.6)	73.5 (21.8)	71.6 (27.3)
Quite tired	2 (11.1)	6 (33.3)	10 (55.6)	A lot of problems	0 (0)	8 (13.3)	9 (12)				
Very tired	0 (0)	7 (30.4)	16 (69.6)	Extreme problems	0 (0)	0 (0)	8 (10.7)				
Annoyed	*n* = 37	*n* = 60	*n* = 73	Feeling worried, sad or unhappy*	*n* = 37	*n* = 59	*n* = 75				
No annoyed	28 (27.5)	32 (31.4)	42 (41.2)	No problems	23 (62.2)	21 (35.6)	21 (28)				
A little bit annoyed	7 (22.6)	11 (35.5)	13 (41.9)	A little bit of problems	10 (27)	20 (33.9)	27 (36)				
A bit annoyed	2 (12.5)	9 (56.2)	5 (31.2)	Some problems	3 (8.1)	9 (15.3)	11 (14.7)				
Quite annoyed	0 (0)	2 (40)	3 (60)	A lot of problems	0 (0)	5 (8.5)	3 (4)				
Very annoyed	0 (0)	6 (37.5)	10 (62.5)	Extreme problems	1 (2.7)	4 (6.8)	13 (17.3)				
Schoolwork/homework	*n* = 36	*n* = 60	*n* = 73	EQ VAS*	*n* = 36	*n* = 58	*n* = 71				
No problems	28 (28.6)	31 (31.6)	39 (39.8)	Mean (SD)	85.7 (17.1)	74.2 (22)	70.6 (29.6)				
A little bit problems	6 (13)	18 (39.1)	22 (47.8)								
A bit problems	1 (8.3)	5 (41.7)	6 (50)								
Quite problems	1 (11.1)	5 (55.6)	3 (33.3)								
Very problems	0 (0)	1 (25)	3 (75)								
Sleep	*n* = 37	*n* = 61	*n* = 74								
No problems	23 (30.7)	24 (32)	28 (37.3)								
A little bit problems	10 (19.2)	23 (44.2)	19 (36.5)								
A bit problems	2 (11.8)	6 (35.3)	9 (52.9)								
Quite problems	1 (8.3)	5 (41.7)	6 (50)								
Very problems	1 (6.2)	3 (18.8)	12 (75)								
Daily routine	*n* = 37	*n* = 60	*n* = 74								
No problems	34 (28.3)	40 (33.3)	46 (38.3)								
A little bit problems	3 (11.1)	11 (40.7)	13 (48.1)								
A bit problems	0 (0)	7 (50)	7 (50)								
Quite problems	0 (0)	1 (25)	3 (75)								
Very problems	0 (0)	1 (16.7)	5 (83.3)								
Able activities	*n* = 37	*n* = 59	*n* = 73								
No problems	28 (28.3)	31 (31.3)	40 (40.4)								
A little bit problems	7 (14.9)	19 (40.4)	21 (44.7)								
A bit problems	1 (8.3)	5 (41.7)	6 (50)								
Quite problems	0 (0)	2 (40)	3 (60)								
Very problems	1 (16.7)	2 (33.3)	3 (50)								

MSK: musculoskeletal; EQ VAS: EQ visual analogue scale; CHU9D: Child Health Utility 9-Dimension; SD: standard deviation; n: number of children and adolescents

Mild pain: 0–3 pain; Moderate pain: 4–6 pain; Severe pain: 7–10 pain on numerical rating scale pain [0–10]

n (%): absolute number and percentage

*: p-value < 0.05 (Chi-square for the descriptive system and t-test for the EQ visual analogue scale)

## Discussion

### Main findings

This was the first study comparing the measurement properties of the EQ-5D-Y-3L, EQ-5D-Y-5L and CHU9D in Brazil. The EQ-5D-Y-3L, EQ-5D-Y-5L, and CHU9D showed poor to moderate test-retest reliability for the descriptive system and moderate to substantial for the EQ VAS in Brazilian children and adolescents with and without self-reported musculoskeletal pain. For children and adolescents with musculoskeletal pain, the EQ-5D-Y-3L and EQ-5D-Y-5L performed adequately according to pre-defined hypotheses for construct validity compared to the PedsQL™. The CHU9D performed adequately according to pre-defined hypotheses for the EQ-5D-Y-5L but not for the EQ-5D-Y-L and PedsQL™. For those without self-reported musculoskeletal pain, only EQ-5D-Y-5L demonstrated adequate construct validity when compared to CHU9D. The EQ-5D-Y-3L, EQ-5D-Y-5L, and CHU9D were all able to discriminate between subsamples of children and adolescents with and without self-reported musculoskeletal pain, whereas the EQ-5D-Y-3L and EQ-5D-Y-5L were also able to discriminate these subsamples according to pain severity.

### Comparison with other studies

Our results demonstrated that the EQ-5D-Y-3L, EQ-5D-Y-5L, and CHU9D were feasible in Brazilian children and adolescents with and without musculoskeletal pain. The findings are similar to other comparative studies of the EQ-5D-Y-3L and EQ-5D-Y-5L, however, the missing data in our study was slightly higher – especially in the ‘doing usual activities’ dimension (12.7%) of the EQ-5D-Y-5L [23, 25, 41, 57]. The higher missing data was 5% in ‘worried’, ‘sad’ and, ‘pain’ dimensions of the CHU9D. These findings were similar to other studies using the EQ-5D-Y-3L, CHU9D, and PedsQL™ with children and adolescents with obesity, functional dyspepsia and other health chronic conditions (i.e., epilepsy, attention deficit hyperactivity disorder) [26, 28, 58]. However, it is important to note that all these studies considered the utility instead of the descriptive system to assess the missing response [26, 28, 58]. Overall, the complete rate of EQ-5D instruments was slightly higher than the CHU9D and PedsQL™. This difference between the complete rate in each of the instruments can possibly be explained as the EQ-5D-Y-3L and EQ-5D-Y-5L are shorter and easier to complete.

The EQ-5D-Y-3L, EQ-5D-Y-5L, and CHU9D presented ceiling effects (considering at least > 15%) for all dimensions and health state profile for both subsamples, except the health state profile of the CHU9D for those with musculoskeletal pain. As we expected, the sample of children and adolescents without self-report musculoskeletal pain presented ceiling effects across the EQ-5D-Y-3L, EQ-5D-Y-5L and CHU9D [41, 49, 59]. Interestingly, from those children and adolescents that self-reported having musculoskeletal pain in the PIP-Kids questionnaire (*n* = 181), 36% did not report any problems in the “pain or discomfort” or “pain” dimensions of the EQ-5D-Y-3L, EQ-5D-Y-5L, and CHU9D, respectively. This could be attributed to the fact that musculoskeletal pain was classified according to self-completed data on presence of pain and not based on a medical diagnosis from a general practitioner or other health professional. The cause of musculoskeletal pain was further heterogeneous in nature. The recall period of the PIP-Kids questionnaire is the ‘last month’ whereas the EQ-5D youth instruments and the CHU9D use ‘today’ [6–8, 33]. Thus, the children and adolescents could have some pain in the last month, but not necessarily on day of the data collection [7, 8, 33]. In addition, children and adolescents with musculoskeletal pain seems to have a significantly high rate of recovery [16]. Finally, the literature support that the EQ-5D with three levels has shown a high ceiling effect in samples with mild and moderate health conditions in adult population [7, 60–62].

The test-retest reliability of the descriptive system of the EQ-5D-Y-3L, EQ-5D-Y-5L, and CHU9D were classified as poor to moderate, indicating inadequate test-retest reliability according to COSMIN criteria. These low to moderate Kappa coefficients were, however, in accordance with our prior hypotheses and similar to other findings in children and adolescents from the general population, including those with chronic conditions (i.e., physical disabilities) [41, 46, 47, 49, 50]. Nevertheless, there are some studies that found an adequate test-retest reliability (Kappa coefficient values > 0.70) for the descriptive system in patients with clinically defined health conditions (i.e., scoliosis idiopathic, major beta-thalassemia, haemophilia, and acute lymphoblastic leukaemia) for the EQ-5D-Y-3L [23, 48, 63, 64] and the CHU9D (osteogenesis imperfecta) [27]. The heterogeneity of children and adolescents with musculoskeletal pain and the self-reporting nature of this pain may have contributed to these results. As we expected, the ICC of the EQ VAS for the EQ-5D-Y-3L and EQ-5D-Y-5L were in accordance with several other studies with results that ranged from moderate to substantial reliability for those children and adolescents from general population and with health chronic conditions (i.e., scoliosis idiopathic, physical disabilities, and chronic kidney disease) [41, 46, 65, 66]. Previous studies with children and adolescents from general population and with osteogenesis imperfecta also demonstrated adequate test-retest reliability (ICC > 0.70) of the CHU9D [27, 49].

In order to reduce the risk of bias for the construct validity, we pre-specified hypotheses with the magnitude and strength of correlations according to the COSMIN recommendations between the EQ-5D-Y instruments, the PedsQL™ and the CHU9D [29, 39], as well as between the CHU9D and the PedsQL™ [27, 28, 49]. Considering the association with the PedsQL™ sub-scores we identified only one study with children and adolescents from general population in Japan, that reached an agreement higher than 75% of hypotheses formulate previously (authors considered correlation [r] > 0.30) [46]. In contrast our findings in those without any musculoskeletal pain showed only 33% agreement of hypotheses. However, we found an adequate construct validity for children and adolescents with musculoskeletal pain with an agreement higher than 75%. Considering the association between the CHU9D and the EQ-5D-Y-3L results from two studies similarly found weak to moderate correlations, however, these studies did not formulate a-priori hypotheses [53, 67]. In this study, the EQ-5D-Y-5L reached more than 75% of pre-specified hypothesis when compared to the CHU9D for both subsamples of children and adolescents with and without musculoskeletal pain. When the CHU9D and the PedsQL™ were compared, we did not reach more than 75% of the hypotheses formulated a-priori for either the subsamples, with or without musculoskeletal pain. In another study that compared the descriptive system of the CHU9D with the scores of the PedsQL™ in children with osteogenesis imperfecta, the authors reached 100% of their hypotheses [27]. The EQ-5D-Y-3L, EQ-5D-Y-5L, and the CHU9D shown good ability to discriminate between those children and adolescents with and without musculoskeletal pain, as reported on PIP-Kids. Furthermore, in those reporting musculoskeletal pain the EQ-5D-Y-3L, EQ-5D-Y-5L, and CHU9D have been able to differentiate between the severity of pain as measured on the NSRP. These findings are in accordance with previous studies using the EQ-5D-Y-3L and EQ-5D-Y-5L descriptive system and the EQ VAS as well as with our a-priori hypothesis [22, 41].

### Strengths and limitations of the study

One of the strengths of this study is that it was conducted following the COSMIN recommendations to compare the measurement properties of the EQ-5D-Y-3L, EQ-5D-Y-5L, and CHU9D [29, 30]. We also assessed preference-based measures of health (supplementary information 1) using a checklist that is used in economic evaluations [31]. We formulated a-priori hypotheses for all the measurement properties tested in this study based on previous studies [27, 28, 41, 46, 49, 50, 53], especially for the construct validity. This study also has some limitations. Although we collected our data in schools, we used the PIP-Kids questionnaire to identify those children and adolescents self-reporting musculoskeletal pain, and we were unable to identify them by a medical diagnostic or International Classification of Diseases – 11th Revision. We evaluated the feasibility of the EQ-5D youth instruments considering the missing data, response, and completion rates according to previous studies [41, 59, 68]. The feasibility did not include the time required to complete the instruments nor assess their comprehensibility with the target population. Furthermore, the instruments of the EQ-5D were randomized, but the descriptive system was answered consecutively, without separation with another task, which could have led to confusion and recall bias. Comparison of HRQoL instruments is challenging due to the differences in recall periods and response scales (e.g., the PedsQL™ uses ‘one month’ and a frequency scale, whereas the EQ-5D-Y-3L, EQ-5D-Y-5L and CHU9D use ‘today’ and a severity scale). In addition, the PedsQL™ domain scores consist of multiple items, which may not directly align with the descriptive systems of the other instruments. Also, hypothesis testing may further influence these results, especially in this study like this one with heterogeneous, and self-reported health condition (e.g. non-specific musculoskeletal pain) [26, 28, 48]. We did not assess other relevant measurements for the generic preference-accompanied measures (i.e., the EQ-5D youth and the CHU9D) such as redistribution properties and discriminatory power (Shannon index) [25, 47]. Another limitation is that there is currently no instrument considered as a gold standard to assess HRQoL for musculoskeletal pain, thus criterion validity could not be assessed. Although the construct validity (according to the COSMIN quality criteria) was assessed, the absence of a gold standard is a barrier for deeply investigation on validity [3, 6, 10, 30, 39]. Furthermore, regarding the results of hypothesis tests for construct validity that did not reach more than 75%, this means that our a-priori hypotheses were not in accordance with the results that we found, not that the instruments have an inadequate construct validity [30].

### Implications for clinicians and policy markers

The EQ-5D-Y-3L, EQ-5D-Y-5L and CHU9D can be used in the clinical practice to understand effects of pain in the HRQoL and self-perceived health status of children and adolescents with and without musculoskeletal pain. The EQ-5D youth instruments and CHU9D have been validated in Brazilian children and adolescents, contribute with researchers to consider it in economic evaluation of clinical trials, especially the EQ-5D-Y-3L value set that was developed in Brazil [35, 69]. Consequently, the EQ-5D-Y-3L value set will support decision making on the incorporation or reimbursement of health technologies in the healthcare system, for Brazilian children and adolescents.

### Unanswered questions and future research

Future studies should test the measurement properties of the EQ-5D youth instruments and CHU9D in other health conditions (i.e., diabetes, asthma, obesity) as well as in different setting (i.e., clinics, hospitals and, community-based population). Future studies should test the measurement properties in children and adolescents under 8 years old using the self-reported and/or proxy-reported versions to understand the feasibility of these instruments in this population. Furthermore, future studies could test the measurement properties of the EQ-5D-Y-3L and CHU9D in Brazilian children and adolescents using the utility score, and investigating the measurement properties regarding the reliability, validity, and responsiveness.

## Conclusion

The EQ-5D-Y-3L, EQ-5D-Y-5L, and CHU9D had poor to moderate test-retest reliability of the descriptive system and moderate levels of reliability of EQ VAS. The EQ-5D-Y-3L and EQ-5D-Y-5L showed adequate construct validity in children and adolescents with musculoskeletal pain. All three instruments showed good known group validity when comparing those with and without musculoskeletal pain. All instruments presented good feasibility and validity and could be used in research and in the clinical practice to assess the HRQoL, especially of children and adolescents with musculoskeletal pain.

## Data Availability

All unanalyzed data included in this study are available upon reasonable request to the study authors and following the ethics recommendations.
